# β-glucuronidase mRNA levels are correlated with gait and working memory in premutation females: understanding the role of *FMR1* premutation alleles

**DOI:** 10.1038/srep29366

**Published:** 2016-07-08

**Authors:** C. M. Kraan, K. M. Cornish, Q. M. Bui, X. Li, H. R. Slater, D. E. Godler

**Affiliations:** 1 School of Psychological Sciences and Monash Institute of Cognitive and Clinical Neurosciences, Monash University, Clayton, Victoria, 3800, Australia; 2 Centre for Molecular, Environmental, Genetic and Analytic Epidemiology, University of Melbourne Carlton, Victoria, 3053, Australia; 3 Cyto-molecular Diagnostic Research Laboratory, Victorian Clinical Genetics Services and Murdoch Childrens Research Institute, Royal Children’s Hospital, Melbourne, Victoria, 3052, Australia; 4 Department of Paediatrics, The University of Melbourne, Melbourne, Victoria, 3052, Australia

## Abstract

Fragile X tremor ataxia syndrome (FXTAS) is a late-onset disorder manifesting in a proportion of *FMR1* premutation individuals (PM: 55-199 CGG triplet expansions). FXTAS is associated with elevated levels of *FMR1* mRNA which are toxic. In this study, relationships between neurocognitive and intra-step gait variability measures with mRNA levels, measured in blood samples, were examined in 35 PM and 35 matched control females. The real-time PCR assays measured FMR1 mRNA, and previously used internal control genes: β-Glucuronidase (*GUS*), Succinate Dehydrogenase 1 (*SDHA)* and Eukaryotic Translation Initiation Factor 4A (*EI4A2*). Although there was significant correlation of gait variability with *FMR1* mRNA levels (*p* = 0.004) when normalized to *GUS (FMR1*/*GUS*), this was lost when *FMR1* was normalized to *SDHA* and *EI4A2* (2IC). In contrast, *GUS* mRNA level normalized to 2IC showed a strong correlation with gait variability measures (*p* < 0.007), working memory (*p* = 0.001) and verbal intelligence scores (*p* = 0.008). PM specific changes in *GUS* mRNA were not mediated by *FMR1* mRNA. These results raise interest in the role of *GUS* in PM related disorders and emphasise the importance of using appropriate internal control genes, which have no significant association with PM phenotype, to normalize *FMR1* mRNA levels.

A number of overlapping mechanisms have been proposed to cause Fragile X-spectrum disorders (FXSD)^1^ involving CGG trinucleotide expansion in the 5′UTR of *FMR1*^2^. These include reduced *FMR1* protein expression (FMRP), elevated levels of non-coding RNA (*FMR4, FMR5, FMR6*)^3^,^4^, mitochondrial dysfunction^5^, CGG repeat-associated non-AUG-initiation^6^, and gain-of-function RNA toxicity^7^. The “gain of function” toxicity^8^ has been one of the more prominent hypotheses used to explain FXSD, where two to eight fold increase in *FMR1* mRNA expression and in levels of its antisense transcript *ASFMR1* have been positively correlated with CGG triplet expansion size^2^. Specifically, RNA from the expanded allele is thought to bind to and sequester mRNA binding proteins from their pre-determined role, leading to dysregulation of multiple neuronal pathways. The primary CGG triplet expansion class associated with this toxicity is known as *FMR1* premutation (PM), ranging between 55 and 199 CGG triplet repeats. This allele class is usually unmethylated within the *FMR1* promoter, is common in the general population (~1 in 150 females and ~1 in 450 males)^9^ and had been associated with two important clinical conditions. Up to 40% of PM males and ~8–16% of PM females develop fragile X-tremor/ataxia syndrome (FXTAS) - a neurodegenerative tremor and ataxia, late-onset adult disorder^10^. An additional ~20% of PM females also develop fragile X-primary ovarian insufficiency (FXPOI), characterised by premature ovarian failure prior to the age of 40^11^.

Furthermore, *FMR1* mRNA silencing and loss of FMRP have been linked to hypermethylated *FMR1* full mutation expansions (FM: >200 CGG triplet repeats), leading to fragile X syndrome - a common neurodevelopmental disorder found ~1 in 4000 in the general population^12^. Up to 40% of all FM individuals have been reported to be either mosaic for methylation and/or CGG size^13^. Because these mosaic individuals have some cells that carry unmethylated FM or PM expansion that over-express *FMR1* and other cells that have methylated FM alleles and under-express *FMR1*, they are also susceptible to disorders associated with *FMR1* mRNA “gain of function” toxicity and FMRP deficiency. In addition, rare cases of males with completely unmethylated FM alleles have been reported that do not have typical FXS (as they express FMRP), but develop a FXTAS phenotype associated with the *FMR1* mRNA toxicity^14^. Therefore, accurate quantification of *FMR1* mRNA levels is of importance for studies of both fragile X syndrome and of other Fragile X-associated Disorders in PM and FM individuals.

Since increase in CGG triplet expansion has been associated with an increase in *FMR1* mRNA toxicity, mitochondrial dysfunction and elevated apoptosis^8^, it is not unreasonable to postulate that CGG triplet repeat size has an impact on the expression profile of many genes. This is consistent with genome wide gene expression analysis showing that multiple pathways are affected by a PM expansion, including glycoprotein biosynthesis, which is mediated through the β-glucuronidase (*GUS*) pathway^15^. An additional interest in investigating GUS mRNA expression in this context is that previous PM studies using reverse transcription real-time quantitative PCR (RT-qPCR) have used this as a single gene for normalisation of *FMR1* mRNA expression (described in Supplementary Table S1).

We postulated that normalising *FMR1* mRNA to *GUS* mRNA levels in blood should impact the correlation that is understood to exist between *FMR1* mRNA levels and PM specific phenotypes, as opposed to the correlation with *FMR1* mRNA expression when normalised to that of other internal control genes. This study, therefore, examined relationships of *FMR1* and *GUS* mRNA levels in blood samples (with reverse transcription real-time quantitative RT-PCR [RT-qPCR] normalized to different internal controls) and gait stepping and working memory measures previously described to reflect PM-specific neurocognitive phenotypes^16^.

## Methods

### Participants

The data for molecular, working memory and gait analyses was collected from 35 PM females and 35 age- and IQ-matched control females previously^16^,^17^, with different components re-analysed to answer questions specific to this study. Groups were matched on height, BMI, age and Wechsler Abbreviated Scale of Intelligence (WASI) Full Scale IQ score (see details in Table 1). Participants were English speaking with no history of epilepsy or of a serious head injury and had normal (or corrected) vision and hearing, and no sign of colour blindness or intellectual disability (as assessed using the WASI Full Scale IQ score <70). The FXTAS Rating Scale was used to screen all participants for features of FXTAS - that is, tremor, ataxia or parkinsonism - or any other neuromotor disorder. A total of 6 PM and 3 control females in this study were taking anti-depressants at the time of testing. One female PM was additionally taking anti-psychotic medication and one other was taking both anti-psychotic and stimulant medication. We did not find any pattern of medication or dietary supplement use by PM or control participants in the first or third quartiles for *GUS* mRNA level (see details in Supplementary Tables S3 and S4). The PM cohort included 28 different pedigrees, including four families of two full blood related sisters, one with three blood related sisters and one with two half-sisters (paternal inheritance). All study participants provided signed informed consent and the study procedures were consistent with the Declaration of Helsinki and approved by the Southern Health Ethics Committee (project 10147B).

### Molecular analyses

CGG sizing and methylation analysis, used to determine activation ratio (AR) were performed on DNA extracted from whole blood. The CGG sizing was performed using the Asuragen AmplideX™ *FMR1* PCR Kit (Asuragen: Austin, TX, USA)^18^. PCR products were assessed via capillary electrophoresis on an Applied Biosytems 3130 Genetic Analyzer with electropherogram analysis conducted using GeneMapper software (Applied Biosystems; Life Technologies, Carlsbad, CA, USA). Methylation sensitive Southern Blot was used to determine AR by targeting a *NruI* restriction site within the *FMR1* CpG island, as previously described^19^. RNA was extracted from peripheral blood mononuclear cells (PBMCs) isolated from whole blood using Ficoll gradient separation^8^. For the *FMR1* mRNA analysis, RT-qPCR on a ViiA™ 7 System (Life Technologies, Global) was used to quantify *FMR1*-5′, *FMR1*-3′, *GUS*, and two internal control genes, *EIF4A2* and *SDHA*, as previously described^20^. The samples were quantified in arbitrary units in relationship to the standard curves performed on each plate. The mean *FMR1* mRNA levels were normalized to: (i) *GUS* alone (*FMR1*/*GUS*); (ii) mean of *EIF4A2*, and *SDHA* mRNA levels (*FMR1*/2IC); (iii) mean of *GUS*, *EIF4A2*, and *SDHA* mRNA levels (*FMR1*/3IC). Other outputs included *GUS* mRNA levels normalized to mean of *EIF4A2* and *SDHA* mRNA levels (*GUS*/2IC), and *EIF4A2* mRNA normalized to *SDHA* mRNA levels (*EIF4A2*/*SDHA*). *EIF4A2* and *SDHA* were chosen as alternative internal control genes as these were the most stably expressed of the gene panel tested using the geNorm approach in another cohort (Supplementary Figure S1).

RNA from each sample was reverse-transcribed in two separate cDNA reactions, with each cDNA analyzed in two separate RT-PCR reactions. The mean of the four arbitrary unit outputs was used as a summary measure for mRNA expression for each participant. Of the 70 blood samples, there were no *FMR1* mRNA results obtained for two PM females and five control females because there was either insufficient RNA extracted or because the results failed the 5′ and 3′ *FMR1* mRNA quality control step^21^.

### Gait and working memory analysis

Spatiotemporal gait characteristics were assessed by a 593 cm long × 89 cm wide instrumented walkway (GAITRite, CIR Systems Inc., Clifton, NJ, USA), as previously described^16^. The GAITRite is a valid and reliable method for assessing spatial and temporal parameters of gait^22^. It has also demonstrated high validity when compared to the ‘gold standard’ 3-dimensional motion analysis system^23^. In accordance with published recommendations, participants traversed the GAITrite walkway across six walking trials per condition (>30 steps per condition), with minimal interruption between walks^24^. Participants initiated and ended walking 1.5 m before and after the mat to reduce the effects of acceleration and deceleration during each walk. A dual-task paradigm that pairs walking with concurrent performance of a cognitive task was employed. This approach induces capacity interference and is commonly used to investigate subtle changes in motor control associated with vulnerabilities in subcortical structures of the brain that would normally be compensated for by higher cortical structures^25^. Gait was assessed under three different dual-task walking conditions: (1) counting backward aloud by 3′s; (2) counting backward aloud by 7′s; (3) finger tapping (i.e., repeating sequence of thumb to finger tapping, moving in the direction from second to fifth digit). Participants were instructed to walk at their preferred speed during all conditions, to not prioritise one task over the other and to keep walking even if they were struggling to identify new word items. The GAITRite computer software recorded average step length and step time for each walk. Step-to-step variability for both variables were calculated as coefficient of variation^1^. Dual-task costs (henceforth referred to as DTCs) were defined as a change in step length variability and step time variability from baseline to dual-task condition. DTC was determined using the following published formula: DTC = [(dual-task score – single-task score)/single-task score] × 100 where a higher value indicated an increase in variability from single to dual-task condition, and a lower value indicated a decrease in variability^26^. The Letter-Number Sequencing (LNS) subtest from the Wechsler Adult Intelligence Scale (WAIS-IV) was selected to assess central executive working memory performance.

### Statistical analyses

For comparison between control and PM groups the distribution of the tested variable was first checked for normal distribution using Shapiro-Wilk test statistics at 5% significance level. All variables that did not satisfy this distribution were transformed using either log or reciprocal function. Generalised estimating (GEE) method was then for the inter-group comparison, taking into account correlation within a family in the PM cohort. Relationship between each molecular variable (outcome) and age or BMI (predictor) in the controls data was assessed using robust regression. This method down weighted effects of outliers that can distort the estimates of regression coefficients. The same method was also used to examine relationships between WASI Full Scale IQ, LNS, DTC step time and length variability (outcome) with each molecular variable. For PM molecular data we initially screened for unusual observations by visual inspection of the scatter plot for outliers, and then performed least square regression and checked observation for high influence and leverage^27^. The outliers were then excluded from analysis using the GEE estimation method. We used p-value < 0.05 as significant level. All analyses were performed using STATA software (version 13.1, StataCorp, College Station, TX, USA).

## Results

### GUS mRNA changes in blood are significantly correlated with gait step length variability in PM females

**Figure 1:**
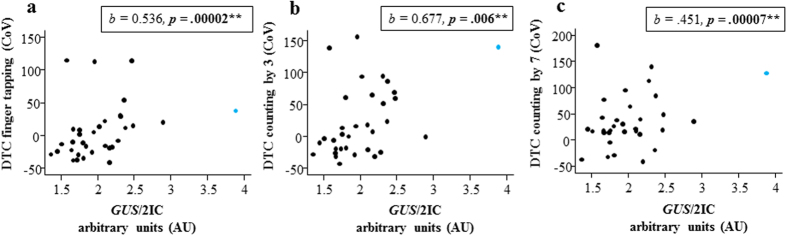
Relationships between *GUS* mRNA normalized to mean expression of *EIF4A2* and *SHDA* (*GUS*/2IC) and different step length variability dual-task cost (DTC) measures in PM females. Scatter plot between *GUS*/2IC and the gait step length variability DTC for **(a)** the finger tapping condition, **(b)** the count by 3′s condition and **(c)** the count by 7′s condition. *Note:* The PM female found to be an outlier for the above relationships is indicated in blue.

In PM females, *GUS/*2IC was significantly correlated with WASI verbal intelligence (*p* = 0.008), working memory (*p* = 0.001) and step length variability DTC for all three conditions: finger tapping (*p* = 0.00002), counting backwards by 3′s (*p* = 0.006) and 7′s (*p* = 0.00007) (Fig. 1, Tables 2 and 3). The ratio between the mRNA levels of two internal control genes used to normalize *GUS* (*SDHA*/*EIF4A2*) was also significantly correlated with working memory (*p* = 0.006) in the PM group (Table 3), indicating that the correlation between working memory and *GUS*/2IC is also affected by changes in expression of the chosen internal control genes *SDHA* and *EIF4A2*. However, there were no correlations between the ratio of these internal control genes (*SDHA*/*EIF4A2*) and step length variability (Tables 2 and 3), which indicated that *GUS*/2IC correlations with the step length variability measure were in fact only due to changes in *GUS* mRNA, and not the internal control genes.

### FMR1 mRNA level correlations with phenotypic measures depend on choice of control genes

Six different methodologies for normalization of *FMR1* mRNA levels in blood were used: *FMR1/GUS*; *FMR1*/2IC; *FMR1*/3IC; *FMR1/GUS* normalized by AR (*FMR1/GUS/AR)*; *FMR1*/2IC normalized by AR *(FMR1*/2IC/AR); *FMR1*/3IC normalized by AR (*FMR1*/3IC/AR). The choice of the normalization method significantly impacted the correlation between *FMR1* mRNA output and clinical outcome variables.

**Figure 2:**
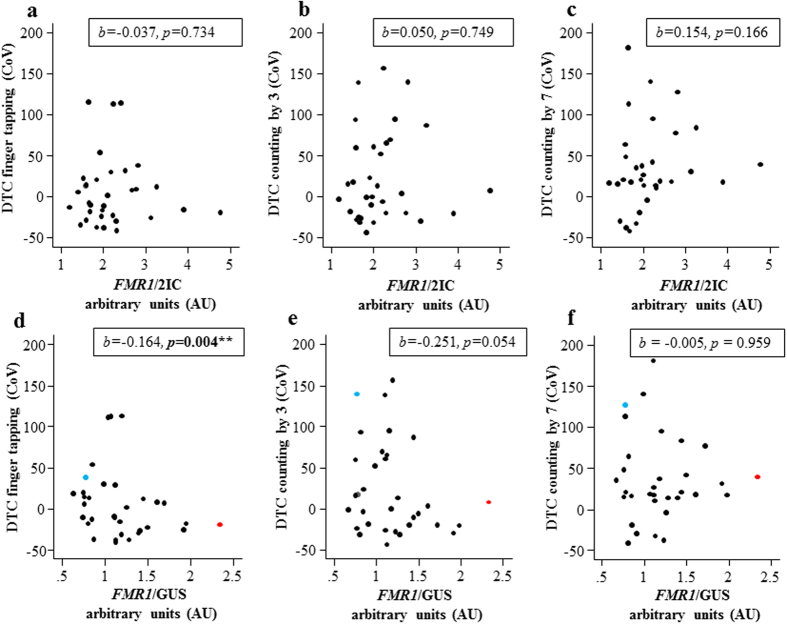
Relationships between *GUS* and *FMR1* mRNA normalized to expression of different internal control genes in PM females. Scatter plot between *FMR1*/2IC and the gait step length variability dual-task cost (DTC) for **(a)** the finger tapping condition, **(b)** the count by 3′s condition and **(c)** the count by 7′s condition. Correlation between *FMR1*/*GUS* and the gait step length variability DTC for **(d)** the finger tapping condition, **(e)** the count by 3′s condition and **(f)** the count by 7′s condition. *Note:* The PM female found to be an outlier for the relationship between gait step length variability DTC and the *GUS*/2IC variable in Fig. 1 is indicated in blue. A PM participant who was an outlier for the relationships between gait step length variability DTC and the *FMR1*/*GUS* variable is highlighted in red.

Correlations between *FMR1*/*GUS* (where *GUS* was the sole internal control) and step length variability were significant for step length variability during the finger tapping condition (*p* = 0.004) and of borderline significance for the counting by 3′s condition (*p* = 0.054) (Fig. 2, Tables 2 and 3). Adjusting for IQ increased the strength of the correlation between *FMR1*/*GUS* and step length variability finger tapping (*p* = 0.005) and counting by 3′s (*p* = 0.022). *FMR1* /*GUS* adjusted for FSIQ was also significantly correlated with poorer performance on the working memory test in PM females (*p* = 0.049) (Table 4). These significant correlations were specific to the PM group, as they were not found in the control group (Supplementary Table S2).

In contrast to *FMR1*/*GUS*, *FMR1*/2IC and *FMR1*/3IC outcomes were not significantly correlated with any gait outcome measures in PM females, suggesting that all positive correlations with *FMR1*/*GUS* were solely driven by changes in *GUS* mRNA levels, rather than that of *FMR1* mRNA. This was also consistent with significant negative spearman’s rank correlation between *FMR1*/*GUS* and *GUS*/2IC, and *GUS*/2IC showing significant correlations with the step length variability measures that were not significantly correlated with *FMR1*/2IC (Fig. 1, Tables 2 and 3). Importantly, *FMR1*/2IC vs *GUS*/2IC outcomes were not significantly correlated with one another (Supplementary Figure S2), suggesting that PM specific changes in *GUS* mRNA were not dependent on PM specific changes in *FMR1* mRNA.

Interestingly, *FMR1*/2IC/AR was significantly correlated with step length variability counting by 3 (*p* = 0.001) and 7 (*p* = 0.001) (Table 3), while *FMR1*/2IC output was not correlated with these measures. Furthermore, the above correlations were not affected after adjustment for FSIQ (Table 4).

## Discussion

*GUS* has been commonly used as an internal control gene for investigations of inter-relationships between gene expression and phenotype due to its stable expression during brain development^28^,^29^ and similar levels of expression in blood to *FMR1*^30^. However, recent studies have found that β-glucuronidase activity can change in response to inflammatory processes, increased body mass index (BMI), older age, gender and insecticide-related neurotoxicity^31^,^32^,^33^. Thus it appears that *GUS* may not be an ideal gene for normalization of *FMR1* mRNA levels, especially if used on its own, as phenotype correlations might be incorrectly attributed solely to changes in *FMR1* mRNA and not *GUS* mRNA levels. Many of the previous studies examining relationships between *FMR1* mRNA toxicity (normalized to *GUS*) and clinical parameters have been based on cohorts with late-onset neurodegenerative disorders or other medical health conditions (e.g. autoimmune disorders) in which *GUS* mRNA changes could be relevant (Supplementary Table S1).

Supplementary Table S1 demonstrates potential significance of the problem, where out of 28 studies examining *FMR1* mRNA levels in FXSD in males and females, 82% (23 out of 28) used GUS as a sole internal control, one study had not stated a normalization strategy used, while only 14% (4 out of 28) used multiple internal control genes to normalize *FMR1* mRNA output data. Some inconsistencies reported include presence of correlations between *FMR1 mRNA* levels and FXDs severity measures in only 46% of the included studies (13 out of 28), with 50% of the studies (14 out of 23) not reporting significant correlations between these measures, and 4% (1 study) examining these relationships, but not stating if they were significant. While the approach to *FMR1* mRNA normalisation is one factor to consider, it must also be highlighted that other issues may be at play. For example, differences in expression of *FMR1* mRNA and internal control genes between tissues, over time and between medications regimes could affect the results. Cohort effects such as recruitment bias, and gender and age differences across studies as well as different phenotype measurements/methodologies are other possibilities.

In this study, while *FMR1* mRNA levels normalized to two internal control genes (*EIF4A2* and *SDHA*) and AR were significantly correlated with step length variability, *FMR1* mRNA levels normalized to only the two internal control genes, and not AR, were not correlated with any clinical variables. This suggests that the significant correlations for *FMR1* mRNA output when normalized for *AR* in this study are driven solely by changes in AR, that may represent X chromosome inactivation skewing, rather than changes in *FMR1* mRNA.

Importantly, this study demonstrates that changes in *GUS* mRNA levels are positively and significantly correlated with gait intra-step variability in adult PM females, and to a lesser degree with poorer performance on working memory and verbal intelligence tests. As expected, this study found that the results of *FMR1* mRNA-phenotype investigations depend on the selection of internal control genes. Most importantly, when *FMR1* mRNA was controlled only by *GUS*, PM females with the highest *GUS* mRNA levels were found to have significantly underestimated *FMR1* mRNA levels. For example, the PM case with the highest *GUS* mRNA level of 3.89 had a low value for *FMR1*/*GUS* (0.78) compared to a moderate value for *FMR1*/2IC (no *GUS*) (2.82) and *FMR1*/3IC *(GUS* and two other genes) (1.48) (Figs 1 and 2). Moreover, *GUS*/2IC was significantly negatively correlated with *FMR1*/*GUS* which again suggests that using *GUS* as a sole internal control gene for the quantification of *FMR1* mRNA introduces variability into the data that is not consistent with changes in *FMR1* mRNA levels. As recently highlighted, this is not a problem unique to the FXSD field, and there is growing recognition that other factors, such as inflammation, can significantly influence expression of internal control genes across various tissues, cell types and clinical manifestations^34^. Indeed, the present study identified that working memory performance in PM females was significantly correlated with both *GUS* mRNA levels and the ratio between the mRNA levels of *EIF4A2* and *SDHA* used to normalize *GUS*, indicating that *GUS* is not likely to be the only gene dysregulated in PM females with working memory deficits.

Abnormal *GUS* mRNA could itself contribute to PM related phenotypes as it encodes the β-glucuronidase enzyme involved in catabolism of glycosaminoglycans (GAGs) within the lysosome^35^. Thus any neuropathology that increases lysosomal activity could also indirectly impact *GUS* transcription. *GUS* mRNA levels may be predictive of FXTAS in PM females, as high gait variability, which has been previously observed in FXTAS, strongly correlated with *GUS* mRNA levels^36^. Furthermore, the scaling and timing of stepping associated with intra-step gait variability is strongly dependent on the same cerebellar structures significantly affected by *FMR1* mRNA toxicity in individuals with the FXTAS phenotype^10^. For this reason, it has previously been argued that PM carriers demonstrating high gait variability could have cerebellar dysfunction and may therefore be either in a pre-clinical stage of FXTAS or have a phenotype that is stable yet reminiscent of the more severe FXTAS^16^.

This study suggests that PM females with high gait variability may also have elevated *GUS* mRNA levels. Furthermore, the lack of significant correlation between *GUS* and *FMR1* mRNA in the present PM group, when normalized to the same internal control genes (*SDHA* and *EIF4A2*) suggests that the PM specific changes in *GUS* expression are not directly mediated by *FMR1* mRNA toxicity. Future studies should explore this lack of association through examining alternative mechanisms/relationships between *GUS* and RAN translation and related sequestration that may occur independent of elevated *FMR1* mRNA transcription. RAN initiated translation of the CGG repeat has been linked to production of a toxic polyglycine protein called FMRpolyG that is associated with activity within the protein degradation pathways^37^, and this may indirectly implicate lysosome enzymes via the process of autophagy previously associated in other settings with abnormal *GUS* expression and neuropathology^38^.

Another mediator that could link changes in GUS mRNA with PM size expansion may be mitochondrial dysfunction, which is itself related to: (i) increased levels of oxidative stress in PM carrier males with FXTAS as well as those with a Parkinsonism phenotype, (ii) elevated *ASFMR1* expression^8^. These findings are relevant to the β-glucuronidase pathway because mitochondrial derived reactive oxygen species (ROS) and ongoing oxidate stress are known putative mechanisms behind abnormal permeability of lysosomal membranes, which has been shown to indirectly effect activity of lysosomal enzymes such as *β*-glucuronidase^39^.

However, while current disease models for PM-related neurotoxicity provide an indirect link to *GUS* gene expression, more research is needed in this area, especially since there are a number of other biological or environment factors that can also effect activity of the β-glucuronidase enzyme^40^,^41^.

The main strength of this study is that it uses previously validated clinical data (working memory and gait analyses) in both PM females and age- and IQ-matched control females^16^,^17^ to shed new light on the PM specific role of *FMR1* expression when normalized to different internal controls. Previous studies, commonly used a single *FMR1* normalization strategy that masked contribution of *GUS*, and this could explain often conflicting evidence on the relationship between *FMR1* mRNA levels and the phenotype (Supplementary Table S1). Another strength is that this study confirms using multiple lines of evidence that measuring mRNA levels with qRT-PCR in PM related disorders requires an improved approach to the internal control methodology that is using at least two or three internal control genes determined to be stably expressed. One important limitation, and the direction for future studies, however, is that, a better approach would be through absolute quantification of mRNA using methods that do not rely on internal control normalization such as competitive PCR^42^ or Droplet Digital PCR^43^, demonstrated to be effective for gene expression analysis in other settings. Other limitations include the relatively small sample size, the need to reproduce these findings in an independent cohort of PM females, and to explore contribution of *GUS* to the phenotype in PM males where FXTAS is more prevalent. Future studies should also examine: (i) *GUS* expression at both mRNA and protein levels in adults with FXSD including FXS, to rule out any associations with FMRP deficit; and (ii) to compare *SDHA* and *EIF4A2* normalization strategy as well as the ratio between the two internal controls across FXSD including FXS.

In summary, this study showed that *GUS* is not an ideal control gene for assessing relationships of *FMR1* mRNA and the PM phenotypes examined in this study. Indeed, previously unrecognised effects from *GUS* may also have played a role in the inconsistencies of previously reported inter-correlations between *FMR1* mRNA and phenotype in PM carriers. The correlations of *GUS* mRNA levels with PM phenotype prompt further investigation of the β-glucuronidase pathway in the pathology of *FMR1* PM-related disorders.

## Additional Information

**How to cite this article**: Kraan, C. M. *et al*. β-glucuronidase mRNA levels are correlated with gait and working memory in premutation females: understanding the role of *FMR1* premutation alleles. *Sci. Rep.* **6**, 29366; doi: 10.1038/srep29366 (2016).

## Supplementary Material

Supplementary Information

## Figures and Tables

**Table 1:** Comparisons between control and PM groups using generalised estimating equations.

	Control (N = 35)		PM (N = 35)		p-value
	Mean	SD	Mean	SD	
Characteristics					
Age	41.11	8.636	41.14	8.335	0.989
BMI	27.28	6.421	27.61	6.338	0.761*
Height	1.652	0.086	1.646	0.072	0.739
IQ test					
FSIQ	111.9	9.541	110.3	10.936	0.506
PIQ	113.9	11.15	110.9	11.136	0.260
VIQ	108.1	11.36	106.6	13.900	0.619
Molecular					
AR	0.575	0.060	0.577	0.053	0.963
*FMR1*/3IC	1.129	0.285	1.655	0.540	10−9**
*FMR1*/2IC	1.422	0.316	2.197	0.751	10−11**
*FMR1*/*GUS*	0.867	0.259	1.190	0.398	10−5*
*GUS*/2IC	1.919	0.429	2.033	0.480	0.318
*SDHA*/*EIF4A2*	1.342	0.207	1.371	0.315	0.803
*FMR1*/3IC/AR	1.978	0.514	2.811	0.923	0.964**
*FMR1*/2IC/AR	2.478	0.528	3.758	1.329	**10**^**−6**^
*FMR1*/GUS/AR	1.523	0.477	2.009	0.664	0.013
*GUS*/2IC/AR	3.346	0.687	3.613	1.012	0.219
*SDHA*/*EIF4A2*/AR	2.375	0.504	2.358	0.608	0.938
Working memory					
LNS	13.89	3.206	12.60	3.474	0.112
Gait step time variability					
DTC Finger tapping	0.425	26.56	4.298	38.76	0.601
DTC Counting by 3	23.36	48.00	40.30	70.16	0.203
DTC Counting by 7	51.52	97.65	66.42	99.64	0.468
Gait step length variability					
DTC Finger tapping	−2.681	32.17	7.216	41.25	0.259
DTC Counting by 3	1.905	33.32	23.51	54.49	**0.036**
DTC Counting by 7	27.50	58.34	38.09	51.92	0.453

*Note*: Mean and SD were presented for raw data; *p*-values computed using *log and **reciprocal transformed. Significant values (*p* < 0.05) in bold.

AR = *FMR1* activation ratio; BMI = Body Mass Index; DTC = Dual Task Cost; *EIF4A2* = Eukaryotic initiation factor 4A-2 mRNA; FSIQ = WASI Full Scale IQ; LNS = Letter number sequencing working memory test; PIQ = WASI Performance IQ; *SDHA* = succinate dehydrogenase complex, subunit A, flavoprotein (Fp) mRNA; VIQ = WASI Verbal IQ.

**Table 2:** PM group *p*-values indicating the strength of correlation between step length variability and molecular parameters using regression method estimated by generalised estimating equations.

	Step length variability DTC Finger tapping			Step length variability DTC Counting by 3			Step length variability DTC Counting by 7		
	*b*	s.e	*p*-value	*b*	s.e	*p*-value	*b*	s.e	*p*-value
*FMR1*/3IC	−0.139	0.077	0.070	−0.104	0.125	0.407	0.074	0.081	0.362
*FMR1*/2IC	0.044	0.082	0.590	0.050	0.155	0.749	0.154	0.111	0.166
*FMR1*/*GUS*	−0.164	0.057	**0.004**	−0.251	0.130	0.054	−0.005	0.099	0.959
*GUS*/2IC	0.536	0.126	**0.00002**	0.677	0.246	**0.006**	0.451	0.113	**0.00007**
*SDHA*/*EIF4A*2	0.139	0.158	0.377	0.062	0.199	0.756	0.139	0.170	0.415
AR	−0.033	0.168	0.843	−0.226	0.181	0.212	−0.193	0.169	0.252
*FMR1*/3IC /AR	−0.009	0.116	0.935	0.140	0.158	0.378	0.177	0.107	0.100
*FMR1*/2IC /AR	0.252	0.141	0.074	0.529	0.156	**0.001**	0.416	0.128	0.001
*GUS*/2IC/AR	0.396	0.141	**0.005**	0.427	0.124	**0.001**	0.325	0.144	**0.024**
*SDHA*/*EIF4A2*/AR	0.066	0.175	0.704	0.100	0.230	0.665	0.136	0.196	0.489

*Note: b* = standardised regression coefficient. Significant values in bold.

AR = *FMR1* activation ratio; DTC = Dual Task Cost; *EIF4A2* = Eukaryotic initiation factor 4A-2 mRNA; *SDHA* = succinate dehydrogenase complex, subunit A, flavoprotein (Fp) mRNA. The *FMR1* mRNA levels were normalized to: (i) *GUS* alone (*FMR1*/*GUS*); (ii) mean of *EIF4A2*, and *SDHA* mRNA levels (*FMR1*/2IC); (iii) mean of *GUS*, *EIF4A2*, and *SDHA* mRNA levels (*FMR1*/3IC); (iv) mean of *EIF4A2*, and *SDHA* mRNA levels divided by AR (*FMR1*/2IC/AR); (v) mean of *GUS, EIF4A2*, and *SDHA* mRNA levels divided by AR (*FMR1*/3IC/AR). The *GUS* mRNA levels were normalized to: (i) mean of *EIF4A2*, and *SDHA* mRNA levels (*GUS*/2IC); (ii) mean of *EIF4A2*, and *SDHA* mRNA levels divided by AR (*FMR1*/2IC/AR). Other outputs included AR used alone, and *EIF4A2* mRNA normalized to *SDHA* mRNA levels (*EIF4A2*/*SDHA*).

**Table 3:** PM group *p*-values indicating the strength of correlation between phenotype and molecular parameters using regression method estimated by generalised estimating equations.

Variable	*FMR1*/3IC	*FMR1*/2IC	*FMR1*/*GUS*	*GUS*/2IC	*SDHA*/EIF4A2*	AR	*FMR1*/3IC/AR	*FMR1*/2IC/AR	*FMR1*/*GUS*/AR	*GUS*/2IC/AR	*SDHA*/*EIF4A2*/AR
*Characteristics*											
Age	0.642	0.630	0.447	0.702	0.472	0.883	0.614	0.607	0.452	0.735	0.456
BMI	0.476	0.487	0.443	0.570	**0.031**	0.366	0.154	0.181	0.124	0.666	0.077
*IQ*											
FSIQ	0.794	0.309	0.873	0.683	0.061	0.128	0.280	0.401	0.769	0.260	0.080
VIQ	0.770	0.237	0.714	**0.008**	0.222	0.988	0.588	0.882	0.680	**0.010**	0.189
PIQ	0.935	0.534	0.638	0.227	0.058	**0.030**	0.338	0.056	0.851	0.200	0.060
*Working memory*											
LNS	0.264	0.492	0.109	**0.001**	**0.006**	0.090	0.441	0.753	0.915	0.105	**0.018**
*Step time variability*											
DTC Finger tapping	0.254	0.562	0.190	0.156	0.794	0.223	0.818	0.908	0.585	0.752	0.911
DTC Counting by 3	0.496	0.952	0.196	0.104	0.762	0.129	0.538	0.220	0.961	0.309	0.996
DTC Counting by 7	0.555	0.415	0.577	0.406	0.458	0.123	0.847	0.465	0.836	0.678	0.188
*Step length variability*											
DTC Finger tapping	0.070	0.734	**0.004**	**0.00002**	0.377	0.843	0.935	0.074	0.107	**0.005**	0.704
DTC Counting by 3	0.407	0.749	0.054	**0.006**	0.756	0.212	0.378	**0.001**	0.545	**0.001**	0.665
DTC Counting by 7	0.362	0.166	0.959	**0.00007**	0.415n	0.252	0.100	**0.001**	0.552	**0.024**	0.489

Significant values (*p* < 0.05) in bold. *SDHA/EIF4A2 was adjusted for BMI in the regression by taking residuals of the regression of *EIF4A2* on BMI.

AR = *FMR1* activation ratio; BMI = Body Mass Index; DTC = Dual Task Cost; *EIF4A2* = Eukaryotic initiation factor 4A-2 mRNA; FSIQ = WASI Full Scale IQ; LNS = Letter number sequencing working memory test; PIQ = WASI Performance IQ; *SDHA* = succinate dehydrogenase complex, subunit A, flavoprotein (Fp) mRNA; VIQ = WASI Verbal IQ.

**Table 4:** PM group *p*-values indicating the strength of correlation between phenotype and molecular parameters adjusted for FSIQ using regression method estimated by generalised estimating equations.

Variable	*FMR1*/3IC	*FMR1*/2IC	*FMR1*/*GUS*	*GUS*/2IC	*SDHA*/EIF4A2*	AR	*FMR1*/3IC/AR	*FMR1*/2IC/AR	*FMR1*/*GUS*/AR	*GUS*/2IC/AR	*SDHA*/*EIF4A2*/AR
*Working memory*											
LNS	0.147	0.955	**0.049**	0.061	**0.032**	0.612	0.098	0.610	**0.011**	0.205	**0.021**
*Step time variability*											
DTC Finger tapping	0.154	0.207	0.188	0.759	0.761	0.432	0.446	0.791	0.403	0.999	0.465
DTC Counting by 3	0.271	0.466	0.196	0.696	0.187	0.476	0.530	0.533	0.398	0.636	0.939
DTC Counting by 7	0.687	0.706	0.528	0.300	0.386	0.197	0.700	0.668	0.993	0.419	0.344
*Step length variability*											
DTC Finger tapping	0.056	0.552	**0.005**	**0.00003**	0.556	0.750	0.854	0.115	0.119	**0.001**	0.809
DTC Counting by 3	0.275	0.821	**0.022**	**0.012**	0.725	0.267	0.703	**0.001**	0.473	**0.003**	0.919
DTC Counting by 7	0.572	0.348	0.984	0.154	0.625	0.184	0.215	**0.001**	0.717	**0.023**	0.529

Significant values (*p* < 0.05) in bold. *SDHA/*EIF4A2* was adjusted for BMI in the regression by taking residuals of the regression of *EIF4A2* on BMI.

AR = *FMR1* activation ratio; BMI = Body Mass Index; DTC = Dual Task Cost; *EIF4A2* = Eukaryotic initiation factor 4A-2 mRNA; FSIQ = WASI Full Scale IQ; LNS = Letter number sequencing working memory test; PIQ = WASI Performance IQ; *SDHA* = succinate dehydrogenase complex, subunit A, flavoprotein (Fp) mRNA; VIQ = WASI Verbal IQ.
