# Supplementary material for: β-glucuronidase mRNA levels are correlated with gait and working memory in premutation females: understanding the role of FMR1 premutation alleles
Source: Sci Rep. 2016 Jul 8;6:29366. doi: 10.1038/srep29366 (PMC4937393; doi:10.1038/srep29366)
Supplement: Supplementary Information [file srep29366-s1.doc]

**Supplementary Data**

**β-glucuronidase mRNA levels are correlated with gait and working memory in premutation females: understanding the role of *FMR1* premutation alleles.**

Kraan CM^1,3^, Cornish KM^1^, Bui QM^2^, Li X^3^, Slater HR^3,4^, Godler DE^3*^

^1^School of Psychological Sciences and Monash Institute of Cognitive and Clinical Neurosciences, Monash University, Clayton, Victoria, 3800, Australia

^2^Centre for Molecular, Environmental, Genetic and Analytic Epidemiology, University of Melbourne Carlton, Victoria, 3053, Australia

^3^Cyto-molecular Diagnostic Research Laboratory, Victorian Clinical Genetics Services and Murdoch Childrens Research Institute, Royal Children’s Hospital, Melbourne, Victoria, 3052, Australia

^4^Department of Paediatrics, The University of Melbourne, Melbourne, Victoria, 3052, Australia

***Corresponding author: david.godler@mcri.edu.au**

**Supplementary Figure S1.** Selection of most stably expressed internal control genes in male PM and control groups using the geNORM approach

**Supplementary Figure S2.** Relationships between *FMR1* mRNA normalized to expression of different sets of internal control genes in the PM group

**Supplementary Table S1:** Summary of human studies that have examined correlations between *FMR1* mRNA in blood and different PM- associated phenotypes

**Supplementary Table S2:** Control group *p*-values indicating the strength of correlation between phenotype and molecular parameters using robust regression

**Supplementary Table S3:** Self-reported medication and supplement use by PM participants found through analysis of *GUS*/2IC quartile output distribution to have the highest and lowest *GUS*/2IC mRNA levels in blood

**Supplementary Table S4:** Self-reported medication and supplement use by control participants found through analysis of *GUS*/2IC quartile output distribution to have the highest and lowest *GUS*/2IC mRNA levels in blood

**Supplementary Figure S1. Selection of most stably expressed internal control genes in male PM and control groups using the geNORM approach.** Determining stability of expression for 7 internal control genes using the geNorm approach^1^ in peripheral blood mononuclear cells (PBMCs) of 58 males with normal size alleles (CGG<40) and 12 males with expanded alleles between 45 and 170 CGG repeats. **(a)** Average expression stability M values, with least to most stable ordered in the left to right direction on the X axis. Both *EIF4A2* and *SDHA* were the most stably expressed genes from the panel tested. **(b)** Variation in average gene expression stability with sequential addition of each internal control gene to the equation (for calculation of the V score normalization factor). In figure (a) the least stably expressed genes are shown on the left side of the X-axis and the most stably expressed genes or combinations of genes are shown on the right side (i.e. *EIF4A2* and *SDHA*). geNorm recommendation is for the V score of 0.15 or below as being sufficiently stable for gene expression normalization in the tested settings. **Note:** All internal control primer/probe mixes (apart from *GUS*) were obtained from PrimerDesign (PerfectProbe ge-PP-12-hu kit) and used at a concentration of 2 μM. Previously published sequences were used for primers and probe for the *GUS* assay^2^.

**
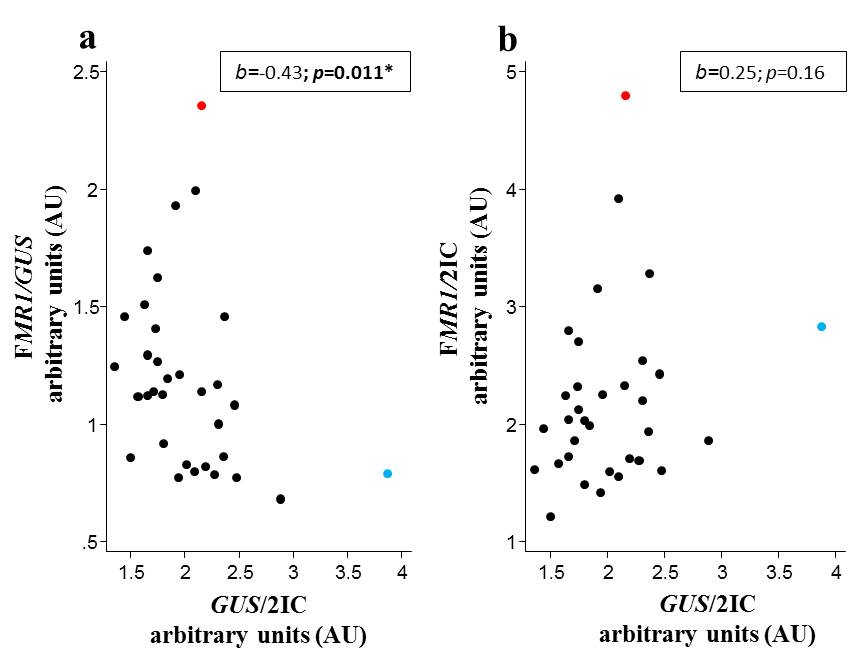
**

**Supplementary Figure S2. Relationships between *FMR1* mRNA normalized to expression of different sets of internal control genes in the PM group.** (a) Correlation between *GUS* mRNA divided by the mean of *SDHA* and *EIF4A2* mRNA (*GUS*/2IC) and *FMR1* mRNA divided by *GUS* mRNA*,* where *GUS* is the sole internal control gene (*FMR1*/*GUS*). **(b)** Correlation between *GUS*/2IC and *FMR1* mRNA divided by the mean of *SDHA* and *EIF4A*2 mRNA (*FMR1*/2IC). *Note*: Outliers for *GUS*/2IC and *FMR1*/*GUS* relationships are highlighted in blue and red, respectively. These outliers are also highlighted using the same colour scheme in Figure 2. *EIF4A2* = Eukaryotic initiation factor 4A-2; *SDHA* = succinate dehydrogenase complex, subunit A, flavoprotein (Fp).

**Supplementary Table S1:** Summary of human studies that have examined correlations between *FMR1* mRNA in blood and different PM- associated phenotypes.

| **[Study ref.]**  **year** | | **Participants** | **Phenotype measurement/methodology** | | | ***FMR1* mRNA data normalisation** | | | | | ***FMR1* mRNA-phenotype correlations in PM group** |  |
| --- | --- | --- | --- | --- | --- | --- | --- | --- | --- | --- | --- | --- |
| ***Significant correlation found between phenotype measurement and FMR1 mRNA (in blood)*** | | | | | | | | | | | |  |
| [3]  2015 | Male: Not included (NI).  Female: FXTAS asymptomatic (FXT-) PM group (n=14) and control group (n=13). | | | | Ocular motor n-back task. | | *FMR1* mRNA levels normalized to average mRNA levels from three internal control genes *(GUS, EIF4A2* and *SDHA).* *FMR1* Activation Ratio (AR) was not measured. | | | *FMR1* mRNA level was significantly correlated with working memory effect scores in the FXT- female PM group. | |  |
| [4]  2015 | Male: NI.  Female: FXT- PM group (n=35) and control group (n=35). | | | | Executive function assessment; self-reported symptoms of attention deficit hyperactivity disorder (ADHD), social anxiety and depression. | | *FMR1* mRNA levels normalized to average of mRNA levels from three internal control genes *(3IC: GUS, EIF4A2* and *SDHA). FMR1* AR determined by methylation sensitive Southern blot and was used to normalize mRNA levels (*FMR1*/3IC/AR). | | | *FMR1* mRNA level was correlated with better working memory and verbal fluency performance in the female FXT- PM group. After normalizaiton of *FMR1* mRNA with AR this relationship was lost. Neither *FMR1* mRNA nor *FMR1*/3IC/AR was found to be a significant predictor of probable dysexecutive/psychiatric disorders in this group. | |  |
| [5]  2014 | Male: NI.  Female: FXT- PM group (n=35) and control group (n=35). | | | | Executive function assessment; self-reported ADHD symptoms; choice step reaction time paradigm. | | *FMR1* mRNA levels normalized to average of mRNA levels from three internal control genes *(GUS, EIF4A2* and *SDHA). FMR1* AR determined by methylation sensitive Southern blot and was used to normalize mRNA levels. | | | *FMR1* mRNA alone was not correlated with any measures in the female FXT- PM group. However, the *FMR1* mRNA controlled by *FMR1* AR was significantly correlated with choice step reaction time performance in this group. | |  |
| [6]  2014 | Male: FXTAS positive (FXT+) PM group (n=30) and control group (n=9).  Female: FXT+ PM group (n=8) and 1 control participant. | | | | Motor section of the Unified Parkinson’s Disease Rating Scale (UPDRS) (except the rigidity item). | | *FMR1* mRNA level normalization method was not stated. *FMR1* AR was not measured. | | | *FMR1* mRNA level was significantly correlated with the body bradykinesia score on the UPDRS and FXTAS clinical stage in a combined group of FXT+ PM males and females. | |  |
| [7]  2013 | Male: FXT+ PM group (n=36), FXT- PM group (n=26) and control group (n=34).  Female: NI. | | | | Diffusion tensor imaging of motor-related fibre tracts. | | *FMR1* mRNA levels normalized to *GUS* mRNA levels. | | | *FMR1* mRNA level was significantly correlated with connectivity strength of the superior cerebellar peduncle in the male FXT+ PM group but not the male FXT- PM group. *FMR1* mRNA level was also significantly correlated with reduced corpus callosum connectivity in the male control group. | |  |
| [8]  2013 | Male: FXT+ PM group (n=29) and control group (n=19).  Female: FXT+ PM group (n=12) and control group (n=13). | | | | Neuropsychological testing of global/general cognitive abilities, executive functioning, attention abilities, and memory; electroencephalogram recording during performance of an auditory oddball paradigm with dual-response requirement. | | *FMR1* mRNA levels normalized to *GUS* mRNA levels. *FMR1* AR was not measured. | | | *FMR1* mRNA level was significantly correlated with reduced P3 amplitude and FXTAS disease stage in a combined group of FXT+ PM males and females. | |  |
| **[Study ref.]**  **year** | **Participants** | | | | **Phenotype measurement/methodology** | | ***FMR1* mRNA data normalisation** | | | ***FMR1* mRNA-phenotype correlations in PM group** | |  |
| [10]  2011 | Male: FXT+ PM group (n=6), FXT- PM group (n=8) and control group (n=7).  Female: FXT+ PM group (n=9), FXT- PM group (n=7) and control group (n=5). | | | | Verbal working memory task performance during functional magnetic resonance imaging (fMRI). | | *FMR1* mRNA levels normalized to *GUS* mRNA levels. *FMR1* AR was not measured. | | | *FMR1* mRNA level was significantly correlated with right ventral inferior cortex activity in a combined group of all PMs, inclusive of FXT- and FXT+ males and females. | |  |
| [11]  2009 | Male: FXT+ PM group (n=34), FXT- PM group (n= 21) and control group (n=30).  Female: FXT+ PM group (n=16), FXT- PM group (n=17) and control group (n=8). | | | | Neuropsychological testing of global/general cognitive abilities; FXTAS rating scale administration; Symptom Checklist-90-Revised (SCL-90-R) assessment of psychiatric symptoms; volumetric magnetic resonance imaging (MRI) of hippocampus. | | *FMR1* mRNA levels normalized to *GUS* mRNA levels. *FMR1* AR determined by methylation sensitive Southern blot and was used to normalize mRNA levels. | | | *FMR1* mRNA level was significantly correlated with left hippocampal volume in the female FXT- PM group. It is not stated whether *FMR1* mRNA was controlled for *FMR1* AR. *FMR1* mRNA level was not significantly correlated with any imaging results in the female FXT+ PM group or either male PM group. | |  |
| [12]  2008 | Male: FXT- PM group (n=11) and control group (n=11).  Female: NI. | | | | Neuropsychological testing of global/general cognitive abilities; SCL-90-R assessment of psychiatric symptoms; volumetric and function MRI investigation of whole brain and hippocampus. | | *FMR1* mRNA levels normalized to *GUS* mRNA levels. | | | *FMR1* mRNA level was significantly correlated with decreased left hippocampal activation and increased right parietal activation in the male FXT- PM group. | |  |
| [13]  2007 | Male: FXT+ PM group (n=36), FXT- PM group (n=25) and control group (n=39).  Female: FXT+ PM group (n=15), FXT- PM group (n=20) and control group (n=11). | | | | Neuropsychological testing of global/general cognitive abilities; structured videotape and neurological rating scale administration; high resolution MRI. | | *FMR1* mRNA levels normalized to *GUS* mRNA levels. *FMR1* AR determined by methylation sensitive Southern blot and was used to normalize mRNA levels. | | | *FMR1* mRNA level was significantly correlated with decreased hippocampal volume in a combined male FXT+ and FXT- PM group. This correlation was also found for *FMR1* mRNA controlled by *FMR1* AR in a combined female FXT+ and FXT- PM group. | |  |
| [14]  2007 | Male: FXT- PM group (n=12) and control group (n=13).  Female: NI. | | | | Neuropsychological testing of global/general cognitive abilities; SCL-90-R assessment of psychiatric symptoms; fMRI paradigm measuring brain response to fearful faces with fear potentiated startle paradigm and measurement of skin conductance during a brief social encounter. | | *FMR1* mRNA levels normalized to *GUS* mRNA levels. | | | *FMR1* mRNA level was significantly correlated with psychiatric symptom severity and right amygdala activation in the male FXT- PM group. *FMR1* mRNA level was not significantly correlated with potentiated startle measures or skin conductance change in this group. | |  |
| [15]  2006 | Male: FXT+ PM group (n=25), FXT- PM group (n=11) and control group (n=21).  Female: NI. | | | | Neuropsychological testing of global/general cognitive abilities; MRI measurement of region brain volumes. | | *FMR1* mRNA levels normalized to *GUS* mRNA levels. | | | In a group that included both male FXT+ and FXT- PM groups *FMR1* mRNA level was significantly correlated with ventricular volume. This correlation was observed for younger males in the combined PM group but not older males in the combined PM group. | |  |
| [16]  2005 | Male: FXT+ PM group (n=42) and FXT- PM group (n=26).  Female: FXT+ PM group (n=22) FXT- PM group (n=122). | | | | Neuropsychological testing of global/general cognitive abilities; SCL-90-R assessment of psychiatric symptoms. | | *FMR1* mRNA levels normalized to *GUS* mRNA levels. *FMR1* AR determined by methylation sensitive Southern blot and was used to normalize mRNA levels. | | | *FMR1* mRNA level was significantly correlated with most SCL-90-R subscales in the male FXT- PM group. *FMR1* mRNA was correlated with anxiety in the female FXT- PM group, but only when the group included PM females with *an* AR determined to be less than 0.5). | |  |
| | **[Study ref.]**  **year** | **Participants** | **Phenotype measurement/methodology** | ***FMR1* mRNA data normalisation** | ***FMR1* mRNA-phenotype correlations in PM group** | | --- | --- | --- | --- | --- |   ***Significant correlation NOT found between phenotype measurement and FMR1 mRNA (in blood)*** | | | | | | | | | | | |  |
| [17]  2015 | Male: NI  Female: PM group (n= 33: 24 of whom had an abnormal neurological exam) and control group (n=13: 3 of whom had an abnormal neurological exam). | | | Structured videotape and neurological rating scale administration; medical health assessment; neuropsychological testing of global/general cognitive abilities; executive function and memory assessment; self-reported anxiety and depression; endocrine measurements. | | | | *FMR1* mRNA levels normalized to *GUS* mRNA levels. *FMR1* AR determined by methylation sensitive Southern blot and to be used for normalization of mRNA levels. | Correlations between phenotype measures and *FMR1* mRNA level are not stated. However, *FMR1* mRNA was found to be not significantly different between females in the PM groups with and without abnormal neurological exam rating scale scores or abnormal endocrinopathy measurements. It is not stated if *FMR1* mRNA was controlled for by AR in the female PM group. | | |  |
| [18]  2015 | Male: FXT+ PM group (n=7), FXT- PM group (n=15) and control group (n=24)  Female: NI. | | | Neuropsychological testing of global/general cognitive abilities; structured videotape and FXTAS rating scale administration; postural sway assessment with sway-metre; structural brain MRI. | | | | *FMR1* mRNA levels normalized to average mRNA levels from three internal control genes *(GUS, EIF4A2* and *SDHA).* | *FMR1* mRNA level was not significantly correlated with postural sway in a male PM group that included both FXT+ and FXT- cases. Correlations are not stated for other measures. | | |  |
| [19]  2015 | Male: FXT- PM group (n=21) and control group (n=20).  Female: NI. | | | Neuropsychological testing of global/general cognitive abilities; working memory and ADHD assessment; temporal attention task. | | | | *FMR1* mRNA levels normalized to *GUS* mRNA levels. AR was not measured. | *FMR1* mRNA level was not significantly correlated with performance on the temporal attention task in the male FXT- PM group. Correlations are not stated for other measures. | | |  |
| [20]  2014 | Male: Child PM group (n=21), child control group (n=16), adult FXT- PM group (n=25) and adult control group (n=30)  Female: Child PM group (n=15), child control group (n=13), adult FXT- PM group (n=43) and adult control group (n=31). | | | Neuropsychological testing of global/general cognitive abilities; simple reaction time task; endogenous/exogenous cueing task. | | | | *FMR1* mRNA levels normalized to *GUS* mRNA levels. AR was not measured. | *FMR1* mRNA level was not significantly correlated with performance on the endogenous/exogenous cueing task in a combined group of all male and female FXT+ and FXT- cases. Correlations are not stated for other measures. | | |  |
| [21]  2014 | Male: FXT- PM group (n=21) and control group (n=22).  Female: NI. | | | Neuropsychological testing of global/general cognitive abilities; executive function and ADHD assessment; ocular motor task (fixation, smooth pursuit, prosaccade, antisaccade). | | | | *FMR1* mRNA levels normalized to *GUS* mRNA levels. | *FMR1* mRNA level was not significantly correlated with ocular motor task performance in the male FXT- PM group. Correlations are not stated for other measures. | | |  |
| [22]  2013 | Male: NI.  Female: FXT+ PM group (n=34) and control group (n=27). | | | Neuropsychological testing of global/general cognitive abilities, executive functioning and memory; electroencephalogram (EEG) recording during performance of a semantic category decision task. | | | | *FMR1* mRNA levels normalized to *GUS* mRNA levels. AR was not measured. | *FMR1* mRNA level was not significantly correlated with neuropsychological performance in the female FXT+ PM group. | | |  |
| [23]  2012 | Male: NI  Female: PM group (n=344: inclusive of FXT+ PMs but proportion not specified) and control group (n=72). | | | Frequency of immune mediated disorders determined via retrospective review of medical records. | | | | *FMR1* mRNA levels normalized to *GUS* mRNA levels. *FMR1* AR determined by methylation sensitive Southern blot and was used to normalize mRNA levels. | *FMR1* mRNA level was not significantly correlated with immune mediated disorders in the female PM group. It is not stated whether *FMR1* mRNA was normalized by AR | | |  |
|  |  | | |  | | | |  |  | | |  |
|  |  | | |  | | | |  |  | | |  |
| **[Study ref.]**  **year** | **Participants** | | | **Phenotype measurement/methodology** | | | | ***FMR1* mRNA data normalisation** | ***FMR1* mRNA-phenotype correlations in PM group** | | | |
| [24]  2011 | Male: NI.  Female: FXT- PM group (n=24) and control group (n=15). | | | Magnitude comparison (distance effect) task. | | | | *FMR1* mRNA levels normalized to *GUS* mRNA levels. *FMR1* AR determined by methylation sensitive Southern blot and was used to normalize mRNA levels. | *FMR1* mRNA level was not significantly correlated with task performance in the female FXT- PM group. It is not stated whether *FMR1* mRNA was normalized by *FMR1* AR. | | |  |
| [25]  2011 | Male: NI.  Female: FXT- PM group (n=30) and control group (n=20). | | | Simple reaction time task. | | | | *FMR1* mRNA levels normalized to *GUS* mRNA levels. *FMR1* AR determined by methylation sensitive Southern blot and was used to normalize mRNA levels. | *FMR1* mRNA level was not significantly correlated with simple reaction time task performance in the female FXT- PM group. | | |  |
| [26]  2011 | Male: NI.  Female: FXT- PM group (n=29) and control group (n=21). | | | Oral motor simple reaction time task and enumeration task. | | | | *FMR1* mRNA levels normalized to *GUS* mRNA levels. *FMR1* AR determined by methylation sensitive Southern blot and was used to normalize mRNA levels. | *FMR1* mRNA level was not significantly correlated with simple reaction time or enumeration task performance in the female FXT- PM group. It is not stated whether *FMR1* mRNA was normalized by *FMR1* AR. | | |  |
| [27]  2011 | Male: FXT+ PM group (n=31), FXT- PM group (n=24) and control group (n=28).  Female: NI. | | | Neuropsychological testing of global/general cognitive abilities and executive function; FXTAS rating scale administration; SCL-90-R assessment of psychiatric symptoms; voxel based morphometry analysis with region of interest for cerebellar regions. | | | | *FMR1* mRNA levels normalized to *GUS* mRNA levels. | *FMR1* mRNA level was not significantly correlated with rating scale scores or brain imaging results in either PM group. Correlations are not stated for other measures. | | |  |
| [28]  2008 | Male: PM group (n=54: inclusive of FXT+ PMs but proportion not specified) and controls (n=51).  Female: PM group (n=82: inclusive of FXT+ PMs but proportion not specified) and controls (n=39). | | | Structured videotape and FXTAS rating scale administration. | | | | *FMR1* mRNA levels normalized to *GUS* mRNA levels. *FMR1* AR determined by methylation sensitive Southern blot and was used to normalize mRNA levels. | *FMR1* mRNA level was not significantly correlated with FXTAS rating scale scores in either male or female PM group. It is not stated whether *FMR1* mRNA normalized by *FMR1* AR in females. | | |  |
| [29]  2005 | Male: FXT+ PM group (n=5), FXT- PM group (n=7) and control group (n=11).  Female: NI. | | | Neuropsychiatric inventory and neuropsychological testing of global/general cognitive abilities; structured videotape and neurological rating scale administration; high resolution MRI. | | | | *FMR1* mRNA levels normalized to *GUS* mRNA levels. | *FMR1* mRNA level was not significantly correlated with task performance or rating scale scores in either male FXT+ or FXT- PM groups. Correlations are not stated for other measures. | | |  |
| [30]  2004 | Male: FXT- PM group (n=20) and control group (n=20).  Female: NI. | | | MRI (grey and white matter). | | | | *FMR1* mRNA levels normalized to *GUS* mRNA levels. | *FMR1* mRNA level was not significantly correlated with any brain imaging measures in the male FXT- PM group. | | |  |
| [31]  2004 | Male: FXT- PM group (n=20) and control group (n=20).  Female: NI. | | | Neuropsychological testing of global/general cognitive abilities, executive functioning, memory, attention, visuospatial processing and language and pragmatics; self-reported symptoms of anxiety, obsessive compulsive disorders and general health. | | | | *FMR1* mRNA levels normalized to *GUS* mRNA levels. | *FMR1* mRNA levels were not significantly correlated with performance on any tasks included in this study in the male FXT- PM group. | | |  |
|  |  | | |  | | | |  |  | | |  |

*Note*: This table demonstrates inconsistencies in methodologies and results of previous studies that have examined *FMR1* mRNA-phenotype correlations in human PM groups. The results for controls are not described in the table unless a significant correlation was found between *FMR1* mRNA and a specific phenotype measure.

1IC = one internal control gene; 3IC = 3 internal control genes; ADHD = Attention deficit hyperactivity disorder; EEG = electroencephalogram; *EIF4A2* = Eukaryotic initiation factor 4A-2 mRNA; fMRI = functional magnetic resonance imaging; *GUS* = *β*-glucuronidase gene; PCR = polymerase chain reaction; MRI = Magnetic resonance imaging; NI = Not investigated; *SDHA* = succinate dehydrogenase complex, subunit A, flavoprotein (Fp) mRNA; SCL-90-R = Symptom Checklist-90-Revised; UPDRS = United Parkinson’s Disease Rating Scale

**Supplementary Table S2:** Control group *p*-values indicating the strength of correlation between phenotype and molecular parameters using robust regression.

| Variable | *FMR1*/  3IC | *FMR1*/  2IC | *FMR1*/  GUS | *GUS*/  2IC | *SDHA*/  *EIF4A2* | AR | *FMR1*/  3IC/AR | *FMR1*/  2IC/AR | *FMR1*/  *GUS*/AR | *GUS*/  2IC/AR | *SDHA*/  *EIF4A2*/AR |
| --- | --- | --- | --- | --- | --- | --- | --- | --- | --- | --- | --- |
| *Characteristics* |  |  |  |  |  |  |  |  |  |  |  |
| Age | 0.304 | 0.151 | 0.512 | 0.199 | 0.312 | 0.198 | 0.329 | 0.058 | 0.622 | 0.061 | 0.516 |
| BMI | 0.677 | 0.769 | 0.344 | 0.080 | 0.072 | 0.256 | 0.691 | 0.399 | 0.379 | **0.017** | 0.236 |
| *IQ* |  |  |  |  |  |  |  |  |  |  |  |
| FSIQ | 0.164 | 0.075 | 0.141 | 0.606 | 0.336 | 0.544 | 0.450 | 0.205 | 0.346 | 0.640 | 0.735 |
| VIQ | 0.364 | 0.154 | 0.287 | 0.641 | 0.769 | 0.460 | 0.729 | 0.671 | 0.800 | 0.626 | 0.708 |
| PIQ | 0.306 | 0.212 | 0.528 | 0.471 | 0.150 | 0.919 | 0.321 | 0.221 | 0.535 | 0.216 | 0.351 |
| *Working memory* |  |  |  |  |  |  |  |  |  |  |  |
| LNS | 0.448 | 0.358 | 0.594 | 0.553 | 0.358 | 0.978 | 0.352 | 0.206 | 0.480 | 0.993 | 0.359 |
| *Step time variability* |  |  |  |  |  |  |  |  |  |  |  |
| DTC Finger tapping | 0.522 | 0.773 | 0.196 | 0.394 | **0.017** | 0.525 | 0.820 | 0.412 | 0.454 | 0.328 | 0.057 |
| DTC Counting by 3 | 0.074 | 0.087 | **0.010** | 0.608 | 0.374 | 0.493 | 0.298 | 0.215 | 0.132 | 0.952 | 0.565 |
| DTC Counting by 7 | 0.969 | 0.788 | 0.816 | 0.806 | 0.309 | 0.531 | 0.828 | 0.530 | 0.682 | 0.549 | 0.569 |
| *Step length variability* |  |  |  |  |  |  |  |  |  |  |  |
| DTC Finger tapping | 0.494 | 0.338 | 0.733 | 0.762 | 0.682 | 0.653 | 0.374 | 0.176 | 0.584 | 0.425 | 0.972 |
| DTC Counting by 3 | 0.292 | 0.493 | 0.332 | 0.452 | 0.965 | 0.797 | 0.268 | 0.413 | 0.255 | 0.219 | 0.932 |
| DTC Counting by 7 | 0.433 | 0.543 | 0.354 | 0.332 | 0.657 | 0.840 | 0.525 | 0.547 | 0.477 | 0.743 | 0.557 |

Significant values (*p* < 0.05) in bold.

AR = *FMR1* activation ratio; BMI = Body Mass Index; DTC = Dual Task Cost; *EIF4A2* = Eukaryotic initiation factor 4A-2 mRNA; FSIQ = WASI Full Scale IQ; LNS = Letter number sequencing working memory test; PIQ = WASI Performance IQ; *SDHA* = succinate dehydrogenase complex, subunit A, flavoprotein (Fp) mRNA; VIQ = WASI Verbal IQ.

**Supplementary Table S3:** Self-reported medication and supplement use by PM participants found through analysis of *GUS*/2IC quartile output distribution to have the highest and lowest *GUS*/2IC mRNA levels in blood

| **PM group cases who have *GUS*/2IC levels in the 4^th^ quartile** | | | |  | **PM group cases who have *GUS*/2IC levels in the 1^st^ quartile** | | | |
| --- | --- | --- | --- | --- | --- | --- | --- | --- |
| Case | *GUS*/2IC mRNA | Medication(s) | Supplement(s) |  | Case | *GUS/*2IC  mRNA | Medication(s) | Supplement(s) |
| 1 | 3.89 | Lovan  Micardis  Estalis continuous  Symbicort Turbuhaler | Caltrate Plus |  | 1 | 1.37 | Lovan | Antihistamine |
| 2 | 2.89 | None | None |  | 2 | 1.45 | Oroxine | Minerals supplement  Fish oil supplement  Probiotic |
| 3 | 2.49 | None | None |  | 3 | 1.52 | None | Dietary supplement |
| 4 | 2.47 | None | Vitamin D supplement  Reproductive system support |  | 4 | 1.58 | None | None |
| 5 | 2.38 | None | Metagenics Insulex  Metagenics Osteo plus management  Silica capsules  Calcium Fluoride  Juice PLUS  Multivitamin  Fish oil supplement |  |  |  |  |  |
| 6 | 2.37 | None | None |  |  |  |  |  |
| 7 | 2.32 | None | None |  |  |  |  |  |
| 8 | 2.31 | None | L-Tyrosine |  |  |  |  |  |
| 9 | 2.29 | None | None |  |  |  |  |  |

**Supplementary Table S4:** Self-reported medication and supplement use by control participants found through analysis of *GUS*/2IC quartile output distribution to have the highest and lowest *GUS*/2IC mRNA levels in blood.

| **Control group cases who have *GUS*/2IC levels in the 4^th^ quartile** | | | |  | **Control group cases who have *GUS*/2IC levels in the 1^st^ quartile** | | | |
| --- | --- | --- | --- | --- | --- | --- | --- | --- |
| Case | *GUS*/2IC mRNA | Medication(s) | Supplement(s) |  | Case | *GUS*/2IC  mRNA | Medication(s) | Supplement(s) |
| 1 | 3.00 | Atacand Plus  Lercanidipine  Metoprolol  Crestor  Escitalopram  Pariet  Doxycycline | Magnesium supplement  Vitamin E supplement  Vitamin B supplement  Fish oil supplement  Multivitamin  Probiotic  Menopausal relief |  | 1 | 1.14 | None | Multivitamin |
| 2 | 2.67 | Symbicort  Neo-Mercazole |  |  | 2 | 1.25 | None | None |
| 3 | 2.64 | None | Multivitamin  Fish oil supplement  Evening primrose oil supplement  Laxative and fiber supplement |  | 3 | 1.50 | None | Vitamin C supplement  Multivitamin  Fish oil supplement  Magnesium supplement |
| 4 | 2.52 | None | None |  | 4 | 1.52 | Mirtazapine  Norimin | None |
| 5 | 2.49 | Nurofen Plus  Naprogesic | None |  | 5 | 1.53 | Trifeme | Multivitamin |
| 6 | 2.48 | None | None |  | 6 | 1.56 | None | Multivitamin |
| 7 | 2.27 | None | None |  | 7 | 1.61 | None | Multivitamin  Omega-3 and Omega-6 Conjugated linoleic acid supplement  Magnesium supplement  Vitamin D supplement  Calcium supplement  Dietary supplement |
| 8 | 2.25 | None | None |  | 8 | 1.63 | None | Multivitamin  Fish oil supplement  Dietary supplement  Herbal medicine |
|  |  |  |  |  | 9 | 1.63 | Microlut | Glucosamine supplement  Calcium supplement  Vitamin D3 supplement |

**Supplementary References**

1. Vandesompele J, De Preter K, Pattyn F, et al. Accurate normalization of real-time quantitative RT-PCR data by geometric averaging of multiple internal control genes. *Genome biology.* 2002;3(7):Research0034.

2. Tassone F, Hagerman RJ, Taylor AK, Gane LW, Godfrey TE, Hagerman PJ. Elevated levels of FMR1 mRNA in carrier males: a new mechanism of involvement in the fragile-X syndrome. *Am J Hum Genet.* 2000;66(1):6-15.

3. Shelton AL, Cornish KM, Godler DE, et al. Delineation of the working memory profile in female *FMR1* premutation carriers: The effect of cognitive load on ocular motor responses. *Behav Brain Res.* 2015.

4. Cornish KM, Kraan C, Bui M, et al. Novel methylation markers of the dysexecutive-psychiatric phenotype in *FMR1* premutation females. *Neurology.* 2015.

5. Hocking DR, Kraan CM, Godler DE, et al. Evidence linking *FMR1* mRNA and attentional demands of stepping and postural control in women with the premutation. *Neurobiol Aging.* 2014.

6. Niu YQ, Yang JC, Hall DA, et al. Parkinsonism in fragile X-associated tremor/ataxia syndrome (FXTAS): Revisited. *Parkinsonism Relat Disord.* 2014;18(14):006.

7. Wang JY, Hessl D, Schneider A, Tassone F, Hagerman RJ, Rivera SM. Fragile X-Associated Tremor/Ataxia Syndrome: Influence of the *FMR1* Gene on Motor Fiber Tracts in Males With Normal and Premutation Alleles. *JAMA Neurol.* 2013;10:1-8.

8. Yang JC, Chan SH, Khan S, et al. Neural Substrates of Executive Dysfunction in Fragile X-Associated Tremor/Ataxia Syndrome (FXTAS): a Brain Potential Study. *Cereb Cortex.* 2013;23(11):2657-2666.

9. Hashimoto R, Srivastava S, Tassone F, Hagerman RJ, Rivera SM. Diffusion tensor imaging in male premutation carriers of the fragile X mental retardation gene. *Mov Disord.* 2011;26(7):1329-1336.

10. Hashimoto R, Backer KC, Tassone F, Hagerman RJ, Rivera SM. An fMRI study of the prefrontal activity during the performance of a working memory task in premutation carriers of the fragile X mental retardation 1 gene with and without fragile X-associated tremor/ataxia syndrome (FXTAS). *Journal of Psychiatric Research.* 2011;45(1):36-43.

11. Adams PE, Adams JS, Nguyen DV, et al. Psychological symptoms correlate with reduced hippocampal volume in fragile X premutation carriers. *Am J Med Genet B Neuropsychiatr Genet.* 2009;153B(3):775-785.

12. Koldewyn K, Hessl D, Adams J, et al. Reduced hippocampal activation during recall is associated with elevated *FMR1* mRNA and psychiatric symptoms in men with the fragile X premutation. *Brain Imaging and Behavior.* 2008;2(2):106-116.

13. Adams JS, Adams PE, Nguyen D, et al. Volumetric brain changes in females with fragile X-associated tremor/ataxia syndrome (FXTAS). *Neurology.* 2007;69(9):851-859.

14. Hessl D, Rivera S, Koldewyn K, et al. Amygdala dysfunction in men with the fragile X premutation. *Brain.* 2007;130(Pt 2):404-416.

15. Cohen S, Masyn K, Adams J, et al. Molecular and imaging correlates of the fragile X-associated tremor/ataxia syndrome. *Neurology.* 2006;67(8):1426-1431.

16. Hessl D, Tassone F, Loesch DZ, et al. Abnormal elevation of *FMR1* mRNA is associated with psychological symptoms in individuals with the fragile X premutation. *Am J Med Genet B Neuropsychiatr Genet.* 2005;139B(1):115-121.

17. Hall D, Todorova-Koteva K, Pandya S, et al. Neurological and Endocrine Phenotypes of Fragile X Carrier Women. *Clin Genet.* 2015.

18. Birch RC, Hocking DR, Cornish KM, et al. Preliminary evidence of an effect of cerebellar volume on postural sway in *FMR1* premutation males. *Genes Brain Behav.* 2015.

19. Wong LM, Tassone F, Rivera SM, Simon TJ. Temporal dynamics of attentional selection in adult male carriers of the fragile X premutation allele and adult controls. *Front Hum Neurosci.* 2015;9:37.

20. Wong LM, Goodrich-Hunsaker NJ, McLennan YA, Tassone F, Rivera SM, Simon TJ. A cross-sectional analysis of orienting of visuospatial attention in child and adult carriers of the fragile X premutation. *Journal of Neurodevelopmental Disorders.* 2014;6(1):45.

21. Wong LM, Goodrich-Hunsaker NJ, McLennan Y, et al. Eye movements reveal impaired inhibitory control in adult male fragile X premutation carriers asymptomatic for FXTAS. *Neuropsychology.* 2014;28(4):571-584.

22. Yang JC, Simon C, Schneider A, et al. Abnormal semantic processing in females with fragile X-associated tremor/ataxia syndrome. *Genes Brain Behav.* 2013;2(10):12114.

23. Winarni TI, Chonchaiya W, Sumekar TA, et al. Immune-mediated disorders among women carriers of fragile X premutation alleles. *Am J Med Genet A.* 2012;10(81):17.

24. Goodrich-Hunsaker NJ, Wong LM, McLennan Y, et al. Young adult female fragile X premutation carriers show age- and genetically-modulated cognitive impairments. *Brain Cogn.* 2011;75(3):255-260.

25. Goodrich-Hunsaker NJ, Wong LM, McLennan Y, et al. Enhanced manual and oral motor reaction time in young adult female fragile x premutation carriers. *J Int Neuropsychol Soc.* 2011;17:1-5.

26. Goodrich-Hunsaker NJ, Wong LM, McLennan Y, et al. Adult Female Fragile X Premutation Carriers Exhibit Age- and CGG Repeat Length-Related Impairments on an Attentionally Based Enumeration Task. *Front Hum Neurosci.* 2011;5(63):14.

27. Hashimoto R, Javan AK, Tassone F, Hagerman RJ, Rivera SM. A voxel-based morphometry study of grey matter loss in fragile X-associated tremor/ataxia syndrome. *Brain.* 2011;134(Pt 3):863-878.

28. Leehey MA, Berry-Kravis E, Goetz CG, et al. *FMR1* CGG repeat length predicts motor dysfunction in premutation carriers. *Neurology.* 2008;70(16 Pt 2):1397-1402.

29. Loesch DZ, Churchyard A, Brotchie P, Marot M, Tassone F. Evidence for, and a spectrum of, neurological involvement in carriers of the fragile X pre-mutation: FXTAS and beyond. *Clin Genet.* 2005;67(5):412-417.

30. Moore CJ, Daly EM, Tassone F, et al. The effect of pre-mutation of X chromosome CGG trinucleotide repeats on brain anatomy. *Brain.* 2004;127(Pt 12):2672-2681.

31. Moore CJ, Daly EM, Schmitz N, et al. A neuropsychological investigation of male premutation carriers of fragile X syndrome. *Neuropsychologia.* 2004;42(14):1934-1947.
